# A Class of Ratio Estimators of a Finite Population Mean Using Two Auxiliary Variables

**DOI:** 10.1371/journal.pone.0089538

**Published:** 2014-02-24

**Authors:** Jingli Lu, Zaizai Yan

**Affiliations:** College of Sciences, Inner Mongolia University of Technology, Hohhot, Inner Mongolia, China; University of Zurich, Switzerland

## Abstract

In sample surveys, it is usual to increase the efficiency of the estimators by the use of the auxiliary information. We propose a class of ratio estimators of a finite population mean using two auxiliary variables and obtain mean square error (MSE) equations for the class of proposed estimators. We find theoretical conditions that make proposed family estimators more efficient than the traditional ratio estimator and the estimators proposed by Abu-Dayeh *et al*. using two auxiliary variables. In addition, we support these theoretical results with the aid of a numerical example.

## Introduction

Use of auxiliary information has been in practice to increase the efficiency of the estimators. Such information is generally used in ratio, product and regression type estimators for the estimation of population mean of study variable. When correlation between study variable and auxiliary variable is positive ratio method of estimation is used. On the other hand if the correlation is negative, product method of estimation is preferred. Some research works have been done in ratio, product and regression type estimators by using an auxiliary variable [1]–[10].

In this study, a class of ratio estimators using two auxiliary variables is considered to estimate a finite population mean for the variable of interest. We considered several special estimators of the suggested estimators. The comparisons between the traditional multivariate ratio estimators and the estimators proposed by Abu-Dayeh *et al*. [11] with the proposed family of estimators using information of two variables are considered. We compared the traditional ratio estimator, the estimators proposed by Abu-Dayeh *et al*. and proposed several special estimators using the statistic data given in Table 1. And we obtained the satisfactory results.

**Table 1:** Data Statistics.

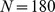	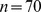	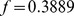	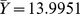
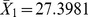	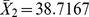	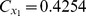	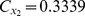
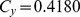	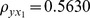	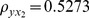	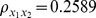
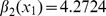	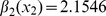		

## Materials and Methods

### The Existed Estimators

The traditional multivariate ratio estimator using information of two auxiliary variables *x*_1_ and *x*_2_ to estimate the population mean,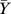, as follows:
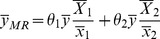
(1)where 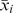 and 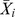
(*i* = 1,2) denote respectively the sample and the population means of the variable *x_i_*; and 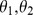 are the weights that satisfy the condition: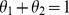 [12].

The MSE of this estimator is given by
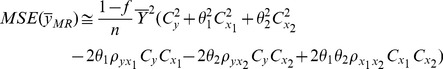
(2)where 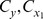 and 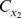 denote the coefficient of variation of *Y*, *X*_1_ and *X*_2_ respectively and 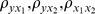 denote the correlation coefficient between *Y* and *X*_1_, *Y* and *X*_2,_ *X*_1_ and *X*_2_ respectively.

The optimum values of 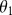 and 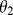 are given by
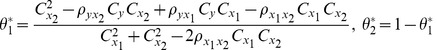


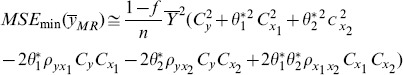
(3)


Abu-Dayeh *et al*. proposed the estimators using two auxiliary variables given by
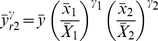
(4)

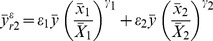
(5)where 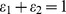.

MSE of these estimators are given as follows:
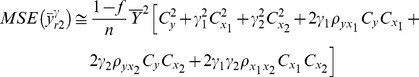
(6)

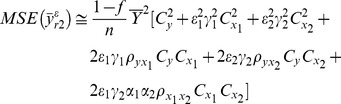
(7)


The optimum values of 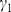 and 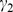 are given by
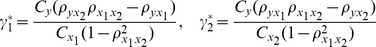


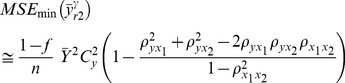
(8)


The optimum values of 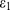 and 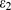 are given by
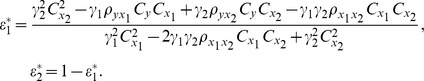


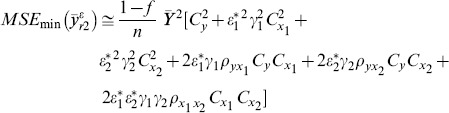
(9)


### The Proposed Family of Ratio Estimators

We propose a class of multivariate ratio estimators using information of two auxiliary variables as follows:
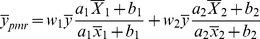
(10)where 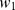 and 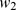 are weights that satisfy the condition: 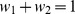, 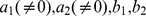 are either real numbers or functions of known parameters.

MSE of these estimators can be found using Taylor series method defined as
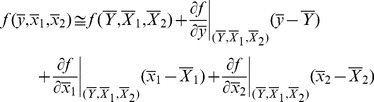
(11)where 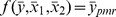


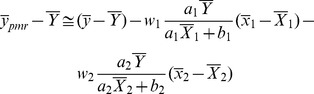


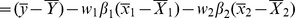
where 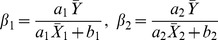


MSE of the class of estimators are given as follows:
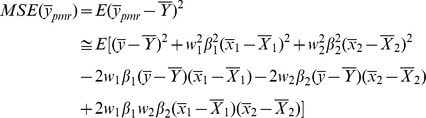


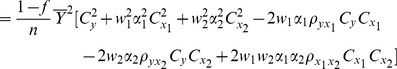
(12)where 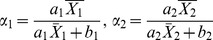


The optimal values of 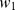 and 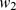 to minimize (12) can easily be found as follows:
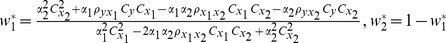


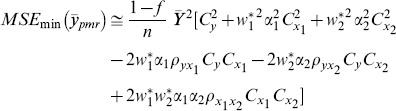
(13)


### Some Members of the Proposed Class of Ratio Estimators 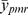


The following are the proposed class of ratio estimators 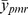:


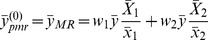
 (The traditional ratio estimator)
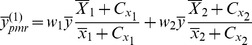


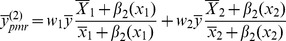


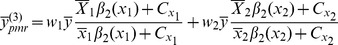


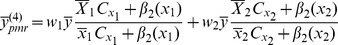


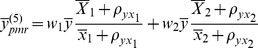


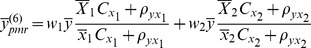


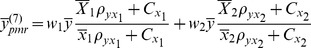


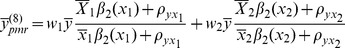


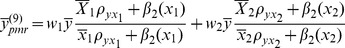



The suitable choices of constants 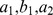 and 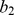 are given in Table 2.

**Table 2:** The suitable choices of constants 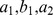 and 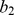.

Estimators	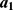	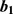	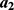	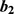
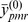	1	0	1	0
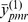	1	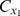	1	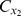
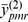	1	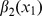	1	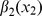
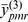	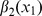	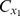	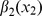	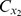
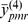	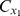	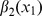	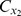	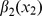
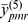	1	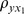	1	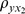
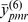	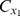	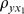	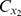	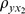
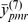	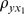	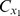	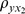	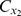
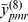	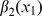	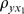	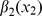	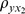
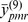	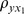	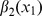	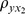	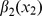

### Efficiency Comparisons

We compare the MSE of the proposed class of ratio estimators given in Eq. (13) with the MSE of the traditional ratio estimator given in Eq.(3)as follows:
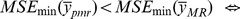


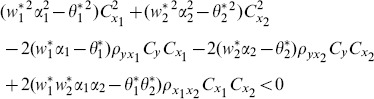
(14)


When this condition is satisfied, the proposed class of ratio estimators 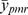 will be more efficient than the traditional ratio estimator.

We compare the MSE of the proposed class of ratio estimators given in Eq. (13) with the MSE of the estimators proposed by Abu-Dayeh *et al*. given in Eq. (8) and Eq. (9) as follows:



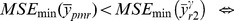


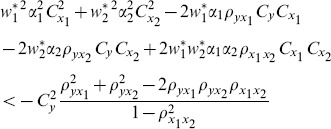
(15)




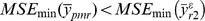


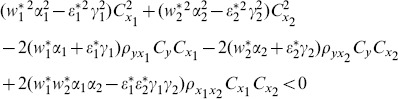
(16)


When these conditions are satisfied, the proposed class of ratio estimators 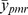 will be more efficient than the estimators proposed by Abu-Dayeh *et al*.

### Numerical Illustration

In this section, we apply the traditional ratio estimator, the estimators proposed by Abu-Dayeh *et al*. and some members of the proposed class of estimators 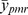, to data whose statistics are given in Table 1 [13]. We assume to take the sample size *n* = 70, from *N* = 180 using SRSWOR. The MSE of these estimators are computed.

## Results and Discussion

MSE of the traditional ratio estimator 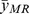, the estimators 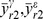 proposed by Abu-Dayeh *et al*. and some members of the proposed ratio estimators 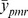 can be seen in Table 3.

**Table 3:** MSE Values of Ratio Estimators.

Estimators	MSE
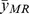	0.157645
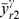	0.157421
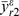	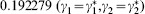
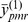	0.157526
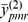	0.157911
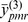	0.157601
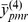	0.162033
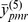	0.157489
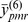	0.157427
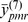	0.157463
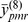	0.157581
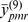	0.159698
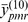	0.157421 ( 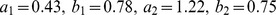 )

From Table 3, we understand that the proposed ratio estimators 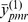, 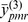, 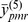, 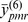, 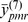, 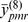 and 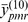 are more efficient than the traditional ratio estimator using two auxiliary variables. When we examine the condition (14), for this data set, we see that all of them are satisfied as follows:

(1) the proposed ratio estimator 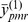


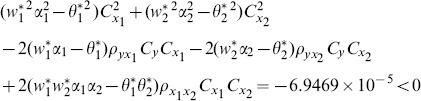



(2) the proposed ratio estimator 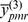


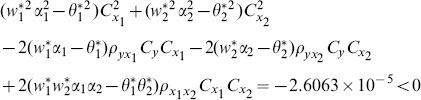



(3) the proposed estimator 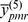


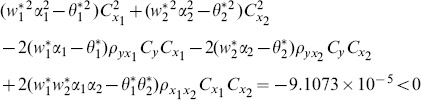



(4) the proposed ratio estimator 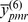


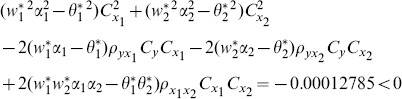



(5) the proposed ratio estimator 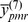.
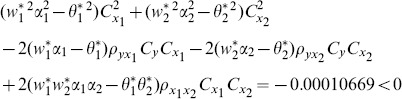



(6) the proposed ratio estimator 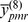


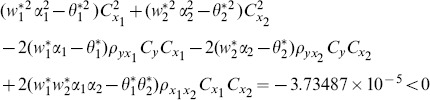



(7) the proposed ratio estimator 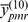


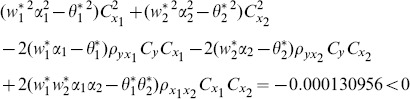



The result shows that the condition (14) is satisfied.

From Table 3, we understand that the efficiency of proposed ratio estimator 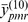 is as good as the estimator 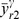 proposed by Abu-Dayeh *et al*. We also understand that the proposed ratio estimators 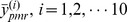 are more efficient than the estimator 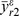 proposed by Abu-Dayeh *et al*. When we examine the condition (16), for this data set, we see that all of them are satisfied as follows:

(8) the proposed ratio estimator 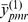


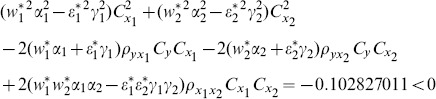



(9) the proposed ratio estimator 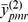


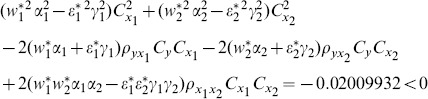



(10) the proposed ratio estimator 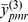


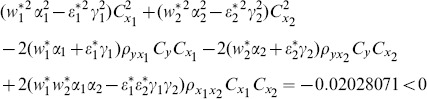



(11) the proposed ratio estimator 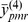


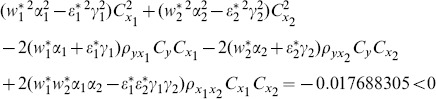



(12) the proposed ratio estimator 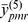


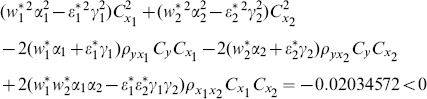



(13) the proposed ratio estimator 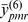


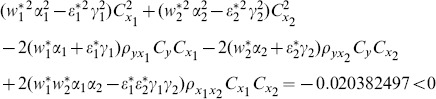



(14) the proposed ratio estimator 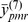


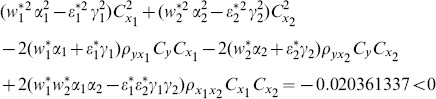



(15) the proposed ratio estimator 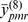


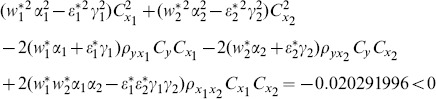



(16) the proposed ratio estimator 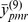


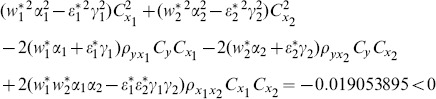



(17) the proposed ratio estimator 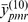


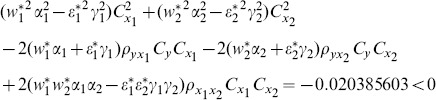



From Table 3, we also understand that the most efficient estimator is the proposed ratio estimator 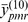 and the estimator 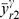 proposed by Abu-Dayeh *et al*. Therefore, we suggest that we should apply the proposed ratio estimator 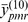 and the estimator 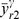 proposed by Abu-Dayeh *et al*. to this data set.

## Conclusions

We develop a class of ratio estimators of a finite population mean using two auxiliary variables and theoretically show that the proposed family ratio estimators are more efficient than the traditional ratio estimator and the estimators proposed by Abu-Dayeh *et al*. in certain conditions. These theoretical conditions are also satisfied by the results of a numerical example.

## References

[1] Choudhury S, Singh BK (2012) A Class of Product-cum-dual to Product Estimators of the Population Mean in Survey Sampling Using Auxiliary Information. Asian J Math Stat 6.

[2] Kadilar C, Cingi H (2004) Ratio estimators in simple random sampling. Appl math comput 151: 893–902.

[3] Kadilar C, Cingi H (2006) An improvement in estimating the population mean by using the correlation coefficient. Hacet J Math Stat 35: 103–109.

[4] Raj D (1965) On a method of using multi-auxiliary information in sample surveys. J Am Stat Assoc 60: 270–277.

[5] Singh H, Tailor R (2003) Use of known correlation coefficient in estimating the finite population mean. Stat Transit 6: 555–560.

[6] Singh HP, Kumar S (2009) A general procedure of estimating the population mean in the presence of non-response under double sampling using auxiliary information. SORT 33: 71–84.

[7] Singh R, Malik S, Chaudhary MK, Verma HK, Adewara A (2012) A general family of ratio-type estimators in systematic sampling. J Reliab Stat Stud 5: 73–82.

[8] Singh R, Malik S, Singh VK (2012) An improved estimator in systematic sampling. J Scie Res 56: 177–182.

[9] Sisodia B, Dwivedi V (1981) A modified ratio estimator using coefficient of variation of auxiliary variable. J Ind Soc Agri Stat 33: 13–18.

[10] Upadhyaya LN, Singh HP (1999) Use of transformed auxiliary variable in estimating the finite population mean. Biometrical J 41: 627–636.

[11] Abu-Dayeh WA, Ahmed MS, Ahmed RA, Muttlak HA (2003) Some Estimators of a Finite Population Mean Using Auxiliary Information. Appl Math Comput 139: 287–298.

[12] Singh D, Chaudhary FS (1986) Theory and analysis of sample survey designs. New Delhi, India: New Age Publication.

[13] Feng SY, Shi XQ (1996) The Sampling Survey–Theory, Method and Practice. Shanghai: Shanghai Scientific and Technical Publishers. (in Chinese)

